# Is the Discount Really Favorable? The Effect of Numeracy on Price Magnitude Judgment: Evidence From Electroencephalography

**DOI:** 10.3389/fnins.2022.817450

**Published:** 2022-06-13

**Authors:** Bijuan Huang, Xiaoyu Liu, Yangyang Wang, Hongxia Li, Jiwei Si, Dawei Wang, Komal Afzal

**Affiliations:** School of Psychology, Shandong Normal University, Jinan, China

**Keywords:** numeracy, price magnitude judgment, promotion frameworks, P3b, alpha desynchronization

## Abstract

Attractive price promotion will induce an unreasonable willingness to purchase, especially through shopping. However, it is not clear how numeracy, one of the essential abilities for understanding and applying numbers, influences the process of purchase judgment. In total, 61 participants were recruited to perform a price promotion task using electroencephalography. The results showed that consumers with low numeracy performed worse than their peers with high numeracy at the behavioral level, and they also had lower P3b amplitude and less alpha desynchronization, regardless of price promotion frameworks. These findings provided evidence on the processing of price information and provided further insights into how numeracy impacts price magnitude judgment.

## Introduction

Shopping online is widespread due to the popularity of the Internet and smart handheld devices. Various price promotion messages are sent by various shopping applications, such as the message “20% off for fashion women’s wear” sent through the Taobao application. Such attractive price promotion messages will increase the consumers’ desire to make a purchase (Feng et al., 2017). In economics and psychology, price cognition plays an important role in consumer behavior models, and subjective judgment of price magnitude is a determining factor for purchase decisions (Monroe, 2003; Winer, 2006; Thomas and Morwitz, 2008; Tang and Song, 2019). However, not all discounts are appropriate for consumers’ decision-making process in a purchase decision context; for instance, an identical product may be sold at two shops with different original prices and discounts. Previous studies have indicated that numeracy, one of the essential abilities for making a rational judgment about the discount, reduces irrational consumption (Tan and Bogomolova, 2016; Feng et al., 2017). However, the cognitive mechanisms and neural underpinnings of how numeracy influences price magnitude judgment remain unknown.

Price contains numerical information represented in the practical context (Cao et al., 2012). Based on the literature on psychological, psycho-physiological, and numerical cognition, the present study aimed to explore and provide further insights into the processing involved in price magnitude judgment and to investigate the effect of numeracy on price magnitude judgment. In particular, this article was organized into three main sections: The first section introduced the concept of price magnitude judgment, two common promotion frameworks, and a putative model of price cognition, which provided a schematic representation of the process of price magnitude judgment put forward by Thomas and Morwitz (2008); informed by this review, the second section outlined the role of numeracy in consumers’ price cognition according to the literature; and the third section present the contributions of this study to the literature on price cognition and the purpose and hypothesis of the present study.

### Price Magnitude Judgment and Promotion Frameworks

In daily purchase scenarios, consumers need to compare prices, such as assessing the difference between the offered price and the reference price of a commodity. This process is referred to as *price cognition*, a cognitive process through which consumers judge the price amount or the difference between two prices and make a price magnitude judgment (Monroe and Lee, 1999; Neidrich et al., 2001; Thomas and Morwitz, 2008; Jones et al., 2012; Raposo et al., 2018).

Two kinds of price promotion frameworks are commonly found in the market to study price magnitude judgment: absolute discount (money-off, e.g., regular price: 52.00 yuan; sale price: 36.50 yuan) and relative discount (percentage-off, e.g., regular price: 52.00 yuan; discount: 70%) (Chen et al., 1998; Jones et al., 2012; Suri et al., 2013; Coulter and Roggeveen, 2014). Purchase behavior will be engendered by such promotions (Feng et al., 2017). Researchers have found that these two kinds of promotion frameworks not only have a positive effect on perceived savings (Krishna et al., 2002) but also influence purchase intention (Chen et al., 1998).

Retailing and marketing studies have revealed the influence of promotion frameworks on price cognition behaviorally and found that various promotion frameworks give rise to different mental representations (Graffeo et al., 2015). Thomas and Morwitz (2008) hypothesized that during the encoding stage of making magnitude judgments between the offered price and reference price, the process of the absolute discount is represented on a mental number line and is compared; the relative discount is represented through arithmetic operations. Most studies in related fields have aimed to reveal the associated framing effects (Graffeo and Bonini, 2018; Guha et al., 2018). However, these two mental representations are different in numerical cognition. Previous studies paid less attention to the different mental representations of various promotion frameworks. Consequently, to understand the psychological mechanisms that underlay consumers’ responses to price, it is better to analyze these two kinds of promotions separately.

Studies on price cognition have also verified findings of magnitude representations in numerical cognition, such as the logarithmic representation of price (Dehaene and Marques, 2002; Furlong and Opfer, 2009) and holistic processing of price comparison (Giuliani et al., 2017; Barone et al., 2020). Drawing lessons from the framework of Dehaene’s (1992) number comparison, Thomas and Morwitz (2008) described the schematic representation of the process of price magnitude judgment. During the encoding stage of making magnitude judgments between the offered price (sale price) and reference price, digit symbols are transcoded into an analog in consumers’ working memory. Then, they can be compared directly by arithmetic operations, or represented on a mental number line and compared. Therefore, the process of price magnitude judgment can be thoughtful and rule-based (as through arithmetic operations) or instinctive and associative (as through analog representations on the mental number line). This offers insights into how consumers represent price information.

### Numeracy and Price Magnitude Judgement

Numeracy refers to the ability to understand and apply numbers (Peters et al., 2007) and includes the ability to understand taxes, perform simple arithmetic operations, and compare numbers (Zhou and Xin, 2012). In the present study, numeracy is referred to as quantitative literacy, namely, applying arithmetic operations and using numerical information in printed materials (Reyna et al., 2009). Numeracy is important because individuals with high numeracy are less constrained by the framework of expression (Peters et al., 2006; Peters and Levin, 2008) and can judge risk information more precisely (Fagerlin et al., 2005; Dieckmann et al., 2009). Graffeo and Bonini (2018) examined the interaction of price presentation formats and numeracy and found that the preferences of individuals with low numeracy changed radically across presentation formats. The authors indicated that processes of evaluating price reductions were modulated by numeracy, and low-skilled consumers were particularly vulnerable to certain forms and depended on a single strategy. Moreover, having good numeracy skills may be particularly important for individuals with low levels of income who need to manage their budgets. Attractive discounts on the surface stimulate consumers’ willingness to purchase and increase sales (Guha et al., 2018). Similar to Black Friday in America, Double 11 is a large-scale promotion event in China. Many unscrupulous businesses increase the original price of the products before this activity starts and then announce the promotion to stimulate consumption. Therefore, it is necessary to understand authentic preferential strength or discount depth. Numeracy here refers to a consumer’s ability to assess whether a price after a discount is an actual bargain (Klichowski and Kroliczak, 2020). Consumers with high numeracy compared prices in a logical manner and successfully met their shopping goals (Tan and Bogomolova, 2016).

Previous studies have consistently found that numeracy moderated the relationship between promotion frameworks and purchase behavior (Kleber et al., 2016; Graffeo and Bonini, 2018; Guha et al., 2018). For example, Graffeo and Bonini (2018) found that consumers with low numeracy were attracted by percentage formats and vulnerable to promotion frameworks. However, the representation of promotion frameworks varied as mentioned earlier. It is better to uncover the cognitive mechanisms of numeracy involved in purchase behavior while controlling for promotion frameworks. At Shop A, jeans is sold at a regular price of 65 with a 20% discount; at Shop B, it is sold at an original price of 85 with a 30% discount. Graffeo et al. (2015) asked consumers to choose the best deal, revealing the mediating role of the decision-making approach used in the relationship between numeracy and choice. They found that consumers with low numeracy used a more incomplete decision-making approach and selected the worst option more often. Consumers with low numeracy regard arithmetic operations as particularly tiring and possess limited numeric ability or have an aversion to using numbers (Graffeo et al., 2015; Kleber et al., 2016). In the schematic representation of the process of price magnitude judgment (Thomas and Morwitz, 2008), these arithmetic operations are critical for the representation of a relative discount. We expected numeracy to affect price magnitude judgment of a relative discount but to have no effect on that of an absolute discount.

The literature on numerical cognition has shown that individual differences in math ability affect numerical representation (e.g., De Smedt et al., 2009; Gómez-Velázquez et al., 2015, 2017) and the processing of arithmetic (e.g., Artemenko et al., 2019; Alnajashi, 2021). For example, De Smedt et al. (2009) found that the distance effect was related to math ability in a numerical comparison task and indicated that high math ability enables a precise representation. Gómez-Velázquez et al. (2015) compared the numerical magnitudes represented in children with different levels of mathematical achievement (low, average, and high achievement) and indicated that math difficulties might be related to a more general magnitude representation problem. Furthermore, the authors (2017) suggested that low-achieving children must exert more memory and attentional effort to meet task demands. Artemenko et al. (2019) recruited individuals with high and low math ability to solve multiplication and division problems and found that individuals with low math ability performed slowly behaviorally and showed less activation in the left supramarginal gyrus (SMG), superior temporal gyrus (STG), and inferior frontal gyrus (IFG) at the neural level. These areas were associated with left perisylvian language areas, which support arithmetic fact retrieval (Klein et al., 2019; Peters and De Smedt, 2018). The authors indicated that individuals with high math ability used more arithmetic fact retrieval while performing an arithmetic task. Alnajashi (2021) found that high performers displayed more alpha power during mental arithmetic tasks. Reyna et al. (2009) argued that difficulties in health decision-making may be due to imprecise representations of number magnitude among those with low numeracy. Based on the aforementioned relevant illustration, we assumed that individuals with low numeracy may perform worse on both tasks and show imprecise representations in the absolute discount task and less efficient processing of arithmetic in the relative discount task.

### The Present Study

Here, we summarized the contributions of this study to the literature on price cognition. This study was intended to explore and provide further insights into the processing of price magnitude judgment from the perspective of numerical cognition. There were two main research orientations on price cognition: numerical cognition and decision-making (Zhou and Xin, 2012). Studies on numerical cognition have considered price cognition as the expansion of numerical cognition and regarded it as situational numerical cognition (Zhou and Xin, 2012). Although the ecological validity of numerical cognition is low, fruitful research and accurate findings on numerical comparison and arithmetic operations in the field of numerical cognition are conducive to a complementary understanding of the findings of price cognition (Zhou and Xin, 2012). Thomas and Morwitz (2008) suggested that the characteristics of price cognition could be understood comprehensively by combining perspectives on numerical cognition and decision-making.

Moreover, a neuroscientific method can enhance the understanding of the marketing and consumer theory (Plassmann et al., 2015). Although the approach is flourishing in consumer neuroscience (Solnais et al., 2013; Plassmann et al., 2015; Bell et al., 2018; Hakim and Levy, 2018), only few researchers attempted to reveal mechanisms that underlie consumers’ responses to price by using high-density electroencephalography (EEG). For example, Jones et al. (2012) investigated how math anxiety affects brain responses to buying decisions and found that for female individuals with high levels of math anxiety, a larger P3 was observed under promotions, which is sensitive to outcome responses in the brain. Consequently, as a unique and efficient gateway, EEG studies provide further evidence for consumers’ responses to price. P3b indexes resource allocation in the temporal–parietal area (Fjell et al., 2007; Polich, 2007; Cao et al., 2012; Jones et al., 2012; Schaefer et al., 2016; Ma et al., 2018; Tang and Song, 2019). Schaefer et al. (2016) indicated that P300 was sensitive to price. In addition, brain oscillation is also an effective means to uncover psychological processes involved in consumer behavior (Plassmann et al., 2015; Bell et al., 2018). The alpha band (8∼12 Hz) is distributed mainly in the posterior area and can be subdivided into the lower alpha (8∼10 Hz) and upper alpha bands (10∼12 Hz) (Artemenko et al., 2019). The alpha band appeared to be important when performing arithmetic tasks (De Smedt et al., 2009; Artemenko et al., 2018, 2019). Alpha desynchronization was related to cognitive resources and the encoding of semantic information (Klimesch et al., 2005). However, EEG evidence for processing price magnitude judgment continues to be lacking.

To minimize the damaging effects of low numeracy, research on how people process numerical information and how such processing can be improved is essential (Reyna et al., 2009). Taken together, the present study investigated the effect of numeracy on price magnitude. Using EEG, this study provided further insights into how numeracy might impact the psychological mechanisms that underlay consumers’ responses to price. In total, 61 freshmen were selected based on screening numeracy to perform a price magnitude judgment task with two kinds of price promotion frameworks, absolute discount and relative discount. Thomas and Morwitz (2008) indicated the differences between processes involved in absolute and relative discount settings during price magnitude judgment tasks. These two frameworks were analyzed separately. At the behavioral level, MANCOVAs were performed for the absolute and relative discount frameworks separately, with numeracy as a between-subject factor and age and gender as covariates. At the neural level, repeated-measures ANCOVAs were performed with numeracy groups as a between-subject factor, electrodes or area as a within-subject factor, and age and gender as covariates. Based on existing research findings mentioned earlier (Artemenko et al., 2019), we hypothesized that low-skilled individuals underperformed high-skilled ones behaviorally, and the low-skilled group would also show smaller P3b amplitude and less alpha desynchronization, especially in the relative discount setting, than high-skilled individuals.

## Materials and Methods

### Participants

This experiment included two experimental groups of 61 freshmen (*M*_*age*_ = 18.23 ± 0.67 years, 30 men) who were selected from a large sample. All students were right-handed and had a normal or corrected-to-normal vision. They had no psychiatric or neurological disorders.

Initially, the students were recruited from a pool of 1,026 students from a public university in China to screen their numeracy ability using a test, the French Kit (French et al., 1963). This test, which is commonly used to measure individuals’ fluency in arithmetic operations, consists of two addition subtests and two subtraction and multiplication subtests. For each subtest, all participants solved problems as quickly and accurately as possible in 2 min. Taking their performance into account, the participants with scores within the first or fifth quintile were assigned to one of two groups. Considering their gender, major, and willingness to participate, 67 subjects were invited to attend subsequent experiments. Among them, two subjects were excluded due to low accuracy in the relative discount task and long reaction times (≥ 3 *SD*) in the absolute discount task, and another four participants were excluded because of excessive artifacts in their EEG data. Finally, 61 participants were included in the reported analysis: 31 in the low-skilled group (73.97 ± 4.67; *M*_*age*_ = 18.26 ± 0.77 years; 16 men) and 30 in the high-skilled group (137.53 ± 12.00; *M*_*age*_ = 18.20 ± 0.55 years, 14 men) (see Table 1). An exploratory study using the same method was conducted behaviorally, and η_*p*_^2^ was valued at 0.17. A desired sample size of 26 was established to detect an expected correlation of 0.20, with 80% power at the 5% significance level. Thus, the sample of the current study was adequate. The two groups differed in numeracy (*p* < 0.001) but not in gender or age (*ps* > 0.05) (see Table 1). All procedures of the study were consistent with the ethical standards of the institutional and/or national research committee and with the 1964 Declaration of Helsinki and its later amendments or comparable ethical standards. The ethics committee of the local university approved the study.

**TABLE 1:** Demographic characteristics in the two groups.

Variables	Low-skilled	High-skilled	Statistical analysis
*N*	31	30	NA
Gender (male/female)	16/15	14/16	χ^2^ = 0.149, *p* > 0.05
Age (*M*/years)	18.26	18.20	
*SD*	(0.77)	(0.55)	*t* = 0.34, *p* > 0.05
range	16∼20	17∼20	
Math ability (*M*)	73.97	137.53	
*SD*	(4.67)	(12.00)	*t* = −27.08, *p* < 0.001
range	16∼83	125∼170	

*M, mean; SD, standard deviation.*

### Procedure

The experiment was conducted in a dark and sound-attenuated room. After the study procedures were explained, the participants provided written informed consent to participate before the experiment started. Then, the participants were seated in comfortable chairs, and stimuli were presented on a monitor located approximately 80 cm in front of them. They were asked to perform a task under two different price promotion frameworks: absolute discount and relative discount. While the task was presented with E-prime 2.0.8.22 (Psychological Software Tools, Pittsburgh, PA) in a blocked fashion, behavioral and EEG data were obtained. The experiment was carried out in two blocks of 88 trials each, including 8 practical trials and 80 experimental trials. The order of the blocks was randomized across participants in an equivalent way for the two groups. After finishing the task, the participants were compensated with a small gift for their participation.

To understand how consumers engage in price magnitude judgment, it is important to consider the reference price, which is used to compare the offered price of a relevant product or service (Neidrich et al., 2001) and indicates whether this price is too high. However, during price magnitude judgment, a large amount of price information that has been experienced in the past can be stored, extracted, and used, which determines the final reference price (Neidrich et al., 2001). Therefore, the reference price will vary according to the context (Adaval and Monroe, 2002), previous experiences (Neidrich et al., 2001), gender (Jones et al., 2012), etc. To control individual differences in the representation of the reference price, we provided a reference price for each commodity related to its actual price in the market to simplify the process of price magnitude judgment.

**FIGURE 1:**
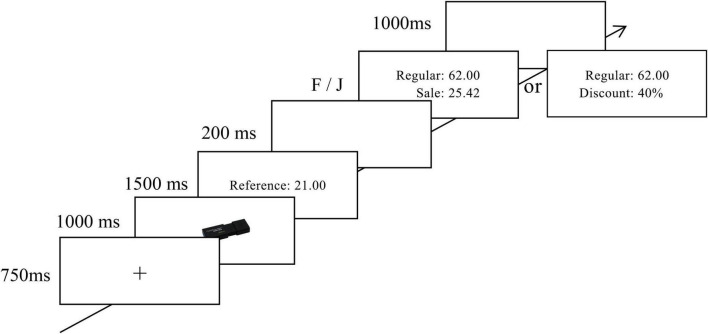
*Illustration of one trial.* Range of reference price, regular price and sale prices are 20 to 100 yuan. And that of discount is 20% to 90%.

Referring to previous studies (Jones et al., 2012; Cao et al., 2015), the specific process proceeded as follows. Each trial began with a fixation cross (750 ms) shown in the center of the screen, followed by the presentation of a picture of merchandise for 1,000 ms, after which the reference price appeared (1,500 ms). Then, a regular price and its sale price or discount appeared, and the participants were asked to decide whether the discounted price (the sale price in the absolute discount setting and the regular price × the discount in the relative discount setting) was higher than the reference price. In the absolute discount setting, the participants were to ignore the regular price and compare the sale and reference prices directly. In the relative discount setting, the participants were to calculate or estimate the result of the regular price and discount and then compare this value to the reference price. During this period, the participants were asked to press the “F” or “J” key on a keyboard as fast as possible to indicate whether the discounted price was less expensive than the reference price. Trials were separated with a black screen shown for 1,000 ms (for the timing of one trial, see Figure 1).

### Electroencephalographic Recordings

EEG data were recorded using a brain production system (BrainVision Recorder 1.20, Brain Products GmbH, Germany),^1^ with a cap of 61 recording electrodes positioned according to the extended 10/10 system and connected to a QuickAmp amplifier. The reference electrode was placed at the midpoint between Fz and Cz, and the ground was located at AFz. Four independent electrodes were also employed. The horizontal and vertical electrooculograms were recorded, with two electrodes placed at the outer canthi and below the right eye, respectively. An additional two electrodes were then placed at the mastoids and used later for referencing. A bandpass filter was set to 0.05–100 Hz, with a sampling rate of 1,000 Hz. The impedances of all electrodes were kept below 10 kΩ.

### Data Analysis

#### Behavioral Data Analysis

Trials with nonresponses, inaccurate responses, or RTs outside ± 3 *SD*s of the individual mean (*M*_*absolute discount*_ = 1.68; *M*_*relative discount*_ = 0.68) were removed before analyses. For accuracy and reaction time, a test of normality was performed in the absolute discount and relative discount frameworks, respectively (skewness: −1.38 to 0.84; kurtosis: 0.15∼2.53). These can be regarded as showing an approximate normal distribution. MANCOVAs were performed for the absolute discount and relative discount frameworks separately, with numeracy as a between-subject factor and age and gender as covariates.

#### Electroencephalography Data Preprocessing and Analysis

For offline signal data analysis, Analyzer 2.1 (BrainVision Analyzer 2.1.0, Brain Products GmbH, German, see text footnote 1) was used. After visual inspection to discard saccades, all offline signals were referred to as the average value of the bilateral mastoids, and the original reference electrode was renamed FCz. The data were digitally filtered (low-pass filter of 35 Hz, zero-phase, and slope of 24 dB/oct). Then, eye-related artifacts, such as blinks, were detected and corrected by independent component analysis. Then, the data were segmented into 1,200-ms epochs that included a 200-ms prestimulus baseline and 1,000 ms after the onset of the promotion in the two different frameworks. In this procedure, trials with correct responses were maintained. After a baseline correction of −200 to 0 ms (with 0 as the timing of promotion presentation) was conducted, epochs containing voltage changes that exceeded ± 80 μV at any electrode were excluded from the analysis. The remaining epochs were separately averaged for each group in each promotion framework (low-skilled group: *M*_*absolute discount*_ = 71.29, *M*_*relative discount*_ = 60.48; high-skilled group: *M*_*absolute discount*_ = 72.17, *M*_*relative discount*_ = 67.07).

**FIGURE 2:**
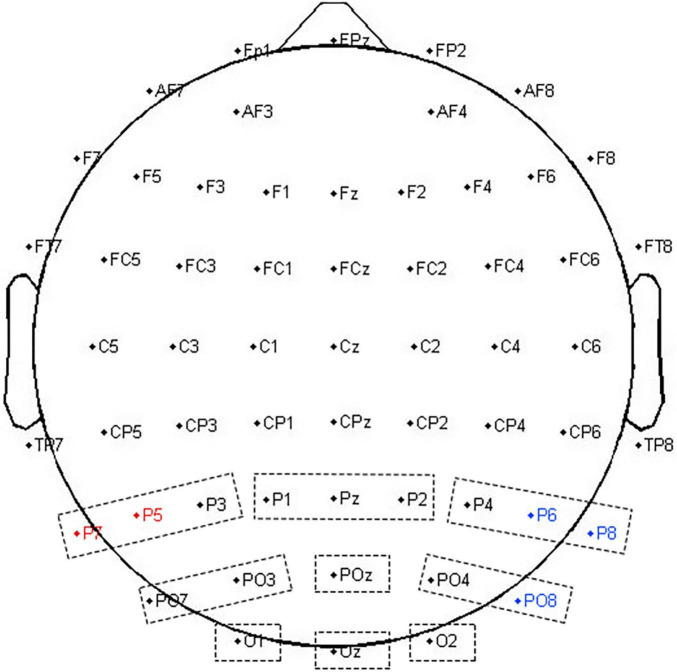
*ROIs in time and time-frequency domain analyses.* Nine dotted rectangles were 9 ROI in the time domain analysis. The red dots represented P5 and P7 electrodes with a significant delayed modulation (both *p*_*cluster*_ < 0.05) in the absolute discount; and the blue dots represented P6, P8, and PO8 electrodes with a significant delayed modulation (both*p*_*cluster*_ < 0.05) in the relative discount in time-frequency domain analysis.

Following the inspection of the grand average ERPs and previous studies, an ERP analysis focused on P3b. In total, nine ROIs (P-left/-central/-right; PO-left/-central/-right; O -left/-central/-right; e.g., P-left included P3, P5, and P7; see Figure 2) were defined. From within-subject averaged waveforms, the peak latency and mean amplitude (±5 time points from peak amplitude) were extracted. The latency and mean amplitude of P3b (250∼500 ms) were quantified as the average over electrodes in each ROI. Then, they were compared using repeated-measures ANCOVAs, with numeracy groups as a between-subject factor, area (parietal, parietal-occipital, and occipital) and hemisphere (left, midline, and right) as within-subject factors, and age and gender as covariates. Greenhouse–Geisser corrections were applied to reduce the type I error rate when Mauchly’s test of sphericity was found to be significant. Bonferroni correction was used for *post-hoc* multiple comparisons.

#### Time–Frequency Domain Analysis

The time–frequency analysis procedure used in the present study drew lessons from the studies of Hu and Iannetti (2019) and Liu et al. (2019). To obtain the time--frequency data of EEG signals from all trials for each participant, a continuous wavelet transform was performed for each electrode using Letswave 7^2^ at 1∼30 Hz. The steps of this transform were set to 100 in the frequency domain. For each subject, the spectrograms across trials were averaged. Then, at each frequency, a baseline correction was applied according to the percentage method: $X_{p}\,\left(t,f\right)=\frac{X\,\left(t,f\right)-R\,\left(f\right)}{R\,\left(f\right)}\times 100\%$, where *X*(*t*,*f*) is the power at time t and frequency f, and *R*(*f*) is the averaged power of frequency f within the reference interval.

A point-by-point two-sample *t*-test combined with a cluster-based permutation test (5,000 times, cluster threshold of 0.05) (Maris and Oostenveld, 2007) was adopted to assess the group-level differences in EEG oscillation power. In the absolute discount setting, significant delayed modulation emerged in the 9∼14 Hz range between 200 and 700 ms, extending over two parietal channels (P5 and P7, both *p*_*cluster*_ < 0.05). In the relative discount setting, significant delayed modulation emerged in the 8∼20 Hz range between 200 and 800 ms, extending over several parietal and parietal–occipital electrodes (P6, P8, and PO8, both *p*_*cluster*_ < 0.05) (see Figure 2). Based on the results of cluster-p and previous studies on numeracy, a significant time–frequency ROI was defined (300∼700 ms, 10∼13 Hz). For each participant, averaged magnitudes in the time–frequency ROI at P5 and P7 for the absolute discount setting and at P6, P8, and PO8 for the relative discount setting were extracted. To reveal the individual differences in the time–frequency domain, the same ANCOVA was performed with numeracy groups as a between-subject factor, electrodes (P5 and P7 for the absolute discount setting and P6, P8, and PO8 for the relative discount setting) as a within-subject factor, and age and gender set as covariates.

## Results

### Low-Skilled Individuals Underperformed Behaviorally

**FIGURE 3:**
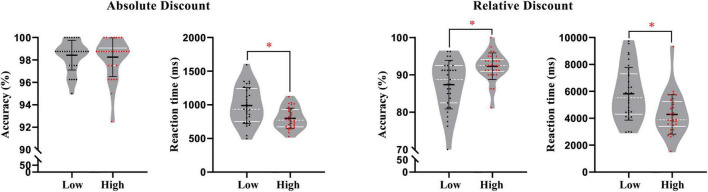
*Behavioral performance in two promotion frameworks.* White lines were quartile of data; black lines were mean and standard deviation of data; each colored dot represented raw data of a subject. *p < 0.05 (the same blow).

A significant effect of numeracy groups was found for reaction times in the absolute discount setting [*F*(1, 57) = 13.30, *p* = 0.001, η*_*p*_*^2^ = 0.19], with low-skilled individuals (989.13 ms) performing slower than their high-skilled peers (799.09 ms) (see Figure 3). There was no significant effect on the percentage of correct responses. In the relative discount setting, regarding the percentage of correct responses and reaction times, the MANCOVA revealed a significant effect of numeracy groups [RT: *F*(1, 57) = 12.35, *p* = 0.001, η*_*p*_*^2^ = 0.18; ACC: *F*(1, 57) = 13.13, *p* = 0.001, η*_*p*_*^2^ = 0.19], showing that the low-skilled group (5818.40 ms; 87.38%) performed worse than the high-skilled group (4284.18 ms; 92.29%) (see Figure 3).

### Lower P3b Amplitudes in the Low-Skilled Group

#### Absolute Discount Setting

For *latency*, the repeated-measures ANOVA showed no significant effects or interactions. For *mean amplitude*, there was a marginally significant effect of numeracy groups [*F*(1, 57) = 3.71, *p* = 0.059, η*_*p*_*^2^ = 0.06] with a smaller P3b amplitude found in the low-skilled group (7.51 μV) than in the high-skilled group (9.31 μV).

#### Relative Discount Setting

**FIGURE 4:**
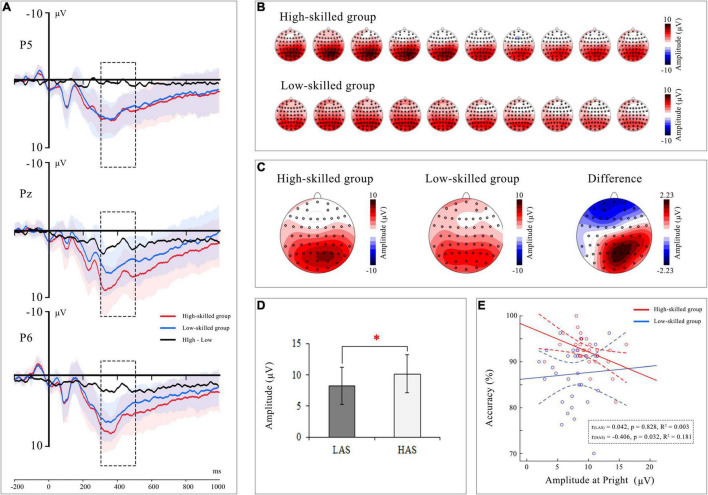
*Group-level P3b waveform (highlighting with dashed rectangle) and topographic map in the relative discount.* **(A)** The waveform of group difference at P3, Pz, and P4 electrodes. And the shaded area represents standard deviation. **(B)** Topographic map of P3b were displayed at the intervals 300 ∼ 500 ms in high-skilled group and low-skilled group. **(C)** The Grand average P3b topographic maps for two experimental groups and their difference. **(D)** Amplitude of P3b reflecting a significant difference for two experimental groups. **(E)** Correlations between P3b amplitude at right parietal area and accuracy. Each colored dot represented raw data of a subject. Full lines represented the best linear fit, and dotted lines represented 95% CI in two experimental groups.

For *latency*, no effects were found. For *mean amplitude*, the main effects of numeracy groups [*F*(1, 57) = 4.29, *p* = 0.043, η*_*p*_*^2^ = 0.07] and interactions of hemisphere × numeracy groups [*F*(2, 114) = 6.58, *p* = 0.002, η*_*p*_*^2^ = 0.10] and area × hemisphere × numeracy groups [*F*(4, 228) = 4.01, *p* = 0.004, η*_*p*_*^2^ = 0.07] reached a significant level. *Post-hoc* analysis showed a more positive P3b amplitude in the high-skilled group than in the low-skilled group at P_*central*_ [*F*(1, 57) = 7.24, *p* = 0.009, η*_*p*_*^2^ = 0.11], P_*right*_ [*F*(1, 57) = 7.55, *p* = 0.008, η*_*p*_*^2^ = 0.12], PO_*central*_ [*F*(1, 57) = 7.02, *p* = 0.010, η*_*p*_*^2^ = 0.11], and O_*right*_ [*F*(1, 57) = 4.65, *p* = 0.035, η*_*p*_*^2^ = 0.08] (see Figure 4). Furthermore, correlation analyses were conducted between RT, ACC, and the significant EEG findings to explore the relationship between neural and behavioral findings, with age and gender set as covariates. The P3b amplitude in the right parietal area [*r*(26) = −0.41, *p* = 0.03] was significantly correlated with accuracy in the high-skilled group (see Figure 4).

### Individual Differences in Alpha Bands

#### Absolute Discount Setting

**FIGURE 5:**
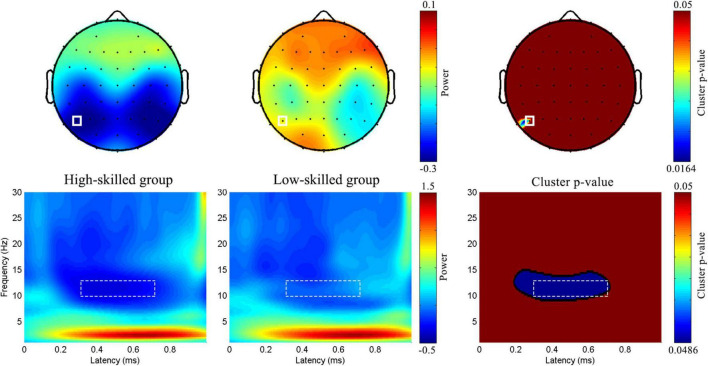
*Group-level topographic map and time-frequency features in the absolute discount.* Topographic maps of the time-frequency ROI was displayed in the first half. And full rectangle represented P5 electrode. The bottom half was the time-frequency features of P5 electrode. Dotted rectangle represented the time-frequency ROI.

Analysis revealed a main effect of numeracy groups [*F*(1, 57) = 13.93, *p* < 0.001, η*_*p*_*^2^ = 0.20; high-skilled: −0.29 ± 0.05 vs. low-skilled: −0.05 ± 0.05; Difference = −0.24, 95% CI [−0.37, −0.11], Cohen’s d = −4.8, power = 0.96] (see Figure 5).

#### Relative Discount Setting

**FIGURE 6:**
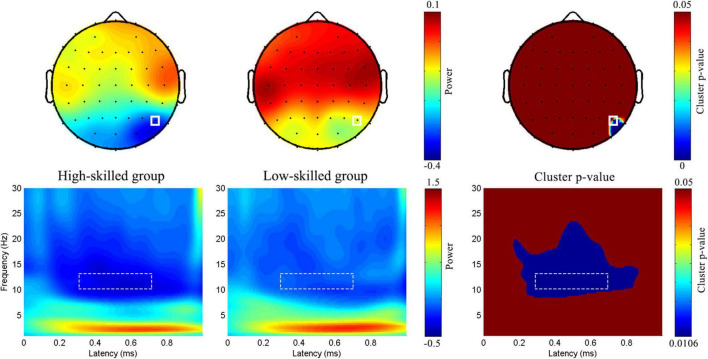
*Group-level topographic map and time-frequency features in the relative discount.* Full rectangle represented P6 electrode.

A significant difference was found between high- (−0.32 ± 0.05) and low-skilled individuals (−0.11 ± 0.05) [*F*(1, 57) = 8.62, *p* = 0.005, η_*p*_^2^ = 0.13; Difference = −0.21, 95% CI [−0.36, −0.07], Cohen’s d = −4.2, power = 0.82] (see Figure 6).

## Discussion

The purpose of the present study was to uncover the cognitive mechanisms and neural basis of how numeracy impacted the process of price magnitude judgment. The main results indicated an effect of numeracy. Specifically, in contrast to high-skilled individuals, their low-skilled peers underperformed behaviorally and showed smaller P3b amplitudes and less alpha desynchronization both in absolute and relative discount settings.

### Price Magnitude Judgment in the Absolute Discount Setting

The findings from the absolute discount setting showed a smaller P3b amplitude and less alpha desynchronization for consumers with low numeracy. Schaefer et al. (2016) indicated that P300 was sensitive to price. P3b indexed resource allocation in the temporal–parietal area (Fjell et al., 2007; Polich, 2007; Cao et al., 2012; Ma et al., 2018; Tang and Song, 2019). Larger amplitude indicated that more attention resources were used and greater interest was elicited (Tang and Song, 2019). Thomas and Morwitz (2008) depicted the process of price magnitude judgment involved in the absolute discount through analog representations on the mental number line. Strong math ability showed a precise representation in the numerical comparison task (De Smedt et al., 2009), which is one of the most commonly used tasks to reveal numerical representation and indicate a mental digital line. Although no effect was observed in terms of accuracy, the results of P3b suggested that consumers with low numeracy paid less attention and displayed a less precise representation even when no arithmetic operations were involved. This was also verified by findings of alpha desynchronization. Alpha desynchronization broadly reflects attention and memory processes (Sun et al., 2020). Larger magnitude indicated that more attentional resources were allocated. Accordingly, due to the representation preciseness relevant to math ability, the influence of numeracy on P3b amplitude and alpha desynchronization showed that compared to consumers with high numeracy, those with low numeracy used less attention resources and displayed an imprecise representation of the price magnitude judgment in the absolute discount setting, consistent with the work of Reyna et al. (2009), who found that difficulties in health decision-making may be due to imprecise representations of number magnitudes among those with low numeracy.

### Price Magnitude Judgment in the Relative Discount Setting

Consistent with other studies, high-skilled individuals outperformed their low-skilled peers behaviorally (e.g., Artemenko et al., 2019). People have to manage various kinds of numerical manipulations, such as paying bills and comparing merchandise sold at different prices in two shops. Accordingly, numeracy is essential for an individual to solve digital problems effectively in daily life. However, why was such a difference found at the behavioral level? Thomas and Morwitz (2008) assumed that in a relative discount setting, the process of price magnitude judgment was thoughtful and rule-based through arithmetic operations. P3b was related to arithmetic and was involved in solving arithmetic problems (Gómez-Velázquez et al., 2015). Results of the present ERP data analysis showed a smaller P3b amplitude for the low-skilled group than for the high-skilled group. As a subcomponent of P300, P3b is an index of cognitive resource allocation (Kok, 2001; Fjell et al., 2007; Polich, 2007). It reflects activity in the temporal–parietal area, which is associated with attention (Polich, 2007) and is also a measure of processing capacity (Kok, 2001). A larger amplitude of P3b indicates more attention devoted to a given task (Salisbury et al., 2001; Tang and Song, 2019). Furthermore, Polich (2007) also summarized that P3b occurred when temporal–parietal memory operations were enhanced by attentional resource activation. The finding for P3b of the current study indicated that consumers with low numeracy allocated fewer attentional resources than their peers with high numeracy.

Correlation analysis results showed that in the high-numeracy group, P3b amplitude negatively correlated with accuracy, which may be related to strategy utilization (Núñez-Peña et al., 2011). Findings from problem-solving consistently showed that high-skilled individuals used strategies that required less cognitive resources, such as fact retrieval, which was stored in long-term working memory (Menon, 2015). Hence, the performance of high-skilled consumers increased as more fact retrieval was used, which consumed less cognitive resources. Traczyk et al. (2018) suggested that individuals with high numeracy should have a broad repertoire of choice strategies and adaptively select these strategies depending on the importance of the given decision. Consumers with low numeracy were apt to adopt a strategy that consumed more resources, such as procedural strategies or exact calculations, to improve performance. Hence, their accuracy increased as P3b amplitude increased, though non-significant. It must be noted that the link between P3b amplitude and performance may vary in relation to numeracy, which needs further research for verification.

Moreover, the two experimental groups varied in the alpha band. Specifically, low-skilled individuals showed less alpha desynchronization relative to their high-skilled peers. This result was consistent with the work of Alnajashi (2021), who found high performers to exhibit larger alpha power during mental arithmetic tasks in P7, corresponding to the left parietal lobe. Upper alpha desynchronization (10∼13 Hz) is mainly distributed in the parietal–occipital area (Artemenko et al., 2019). Decreases in upper alpha desynchronization improved behavioral performance and reflected reduced demands on cognitive resources (De Smedt et al., 2009). In the present study, consistent with the findings for P3b, individual differences were observed while judging price magnitude; the low-skilled group consumed more cognitive resources than the high-skilled group.

What is more interesting is that lateralization was observed in the EEG analysis of alpha desynchronization. The electrodes of interest were P5 and P7 in the absolute discount setting and P6, P8, and PO8 in the relative discount setting. According to Koessler et al. (2009), the P5 and P7 electrodes correspond to regions of the left middle temporal gyrus and left inferior temporal gyrus, respectively; the P6, P8, and PO8 electrodes correspond to regions of the right middle temporal gyrus, right inferior temporal gyrus, and right middle occipital gyrus in anatomical location, respectively. A schematic circuit diagram of basic neurocognitive processes involved in arithmetic indicates that the number form was decoded in the core dorsal parietal cortex and ventral temporal–occipital cortex and represented numerical quantity visuospatially together with the intraparietal sulcus (Menon, 2015; Price et al., 2018). In the present study, regardless of the left and right temporal–occipital areas, less alpha desynchronization was observed in low-skilled individuals, which was consistent with the negative relationship between the resting-state connection of the medial temporal lobes and IPS and the development of math skills (Price et al., 2018).

Although promotions can induce purchase willingness and price is also a determining factor involved in purchase decisions, the present study did not address purchase behaviors, such as purchase willingness, directly. Additionally, as mentioned before, the ecological validity of studies on numerical comparison and arithmetic operation is low in the field of numerical cognition. Further studies should address this question by designing experimental tasks that are more in line with the actual buying environment. For example, on websites, identical merchandise is sold at different stores with different promotions. Using the eye-tracking technique, eye movements of the browsing process can be recorded with a focus on landing positions, first fixation durations, and numbers of fixations to uncover the association between numeracy and price magnitude judgment.

## Conclusion

During times of economic depression, lower product prices are indeed more attractive to consumers. Because of the prevalence of the Internet, consumers receive various price promotion messages. The prices of an identical product may be varied across online stores. From the perspective of numerical cognition, the present study revealed the role of numeracy in consumers’ price magnitude judgment and found consumers with low numeracy to underperform behaviorally (lower accuracy and longer reaction times). During price processing, these individuals represented numerical magnitude less precisely and used less attentional resources (smaller P3b amplitudes and less alpha desynchronization) in the late evaluation stage. The findings of the present study provide further evidence for understanding how numeracy may impact the representation of the process of price magnitude judgment and extend our knowledge of the neural processing of price. This work also has practical implications for preventing an unreasonable willingness to purchase engendered by promotions and for identifying optimal merchandise.

## Data Availability Statement

The raw data supporting the conclusions of this article will be made available by the authors, without undue reservation.

## Ethics Statement

The studies involving human participants were reviewed and approved by the Ethics Committee of the Shandong Normal University. The patients/participants provided their written informed consent to participate in this study.

## Author Contributions

JS and BH designed and conceptualized the study. BH performed the research, analyzed the data, and wrote the main manuscript. XL, YW, HL, and DW edited and revised the manuscript. KA provided some important suggestions on data analyses and manuscript writing. All authors reviewed the manuscript.

## Conflict of Interest

The authors declare that the research was conducted in the absence of any commercial or financial relationships that could be construed as a potential conflict of interest.

## Publisher’s Note

All claims expressed in this article are solely those of the authors and do not necessarily represent those of their affiliated organizations, or those of the publisher, the editors and the reviewers. Any product that may be evaluated in this article, or claim that may be made by its manufacturer, is not guaranteed or endorsed by the publisher.

## Footnotes

1
http://www.brainproducts.com

2
https://www.letswave.org
